# Plant and Trees Pathogens: Isolation, Characterization and Control Strategies (1.0)

**DOI:** 10.3390/jof9040416

**Published:** 2023-03-28

**Authors:** Salah-Eddine Laasli, Essaid Ait Barka, Rachid Lahlali

**Affiliations:** 1 Phytopathology Unit, Department of Plant Protection, Ecole Nationale d’Agriculture de Meknès, Km 10, Rte Haj Kaddour, BP S/40, Meknes 50001, Morocco; laaslisalaheddine@gmail.com; 2 Research Unit Induced Resistance and Plant Bioprotection, USC INRAe 1488, SFR Condorcet FR CNRS 3417, Faculty of Sciences, University of Reims Champagne-Ardenne, 51687 Reims, France

Agricultural production is under constant threat from biotic and abiotic stresses. Among biotic factors, pathogens account for substantial crop losses worldwide. Wheat and barley, as staple crops, are no exception, with losses ranging from 30 to 70% annually [1]. Additionally, grapevine trunk diseases (GTDs) have been a significant threat to the viticulture industry, causing yield losses and decreasing grapevine quality [2]. In recent years, the use of beneficial bacteria in plant disease management has gained attention as a promising sustainable and eco-friendly alternative to chemical-based treatments [3]. This article provides an overview of the beneficial bacteria used for cereal-crop protection against wheat and barley pathogens, and their potential application in GTD management.

Beneficial bacteria have been demonstrated to protect wheat and barley against major fungal pathogens [3]. In wheat, *Bacillus* strains have been found to reduce the disease incidence of *Fusarium graminearum* and *Zymoseptoria tritici*, both responsible for significant economic losses in the context of wheat production. The efficacy of *Bacillus* strains in regulating these pathogens is attributed to the production of secondary metabolites that mediate direct antagonism and induce plant resistance against the pathogens. Similarly, in barley, *Pseudomonas*, *Bacillus* and *Paraburkholderia* spp. have been reported to be effective against *Pyrenophora teres*, a pathogen responsible for significant yield losses [4]. The mode of action of beneficial bacteria/molecules is still not fully understood, and further research is required to enhance the solution-based crop protection strategy [5].

GTD is caused by various fungal pathogens, with the most common being *Botryosphaeriaceae*, *Phaeoacremonium*, and *Eutypa*. According to Kenfaoui et al. [6], the use of beneficial bacteria in GTD management has been limited, but recent studies have shown promising results. Endophytic bacteria, such as *Bacillus*, *Pseudomonas*, and *Streptomyces* spp., have been shown to colonize grapevine tissues, inhibiting the growth of fungal pathogens and inducing plant defense mechanisms [7,8]. The molecular mechanisms involved in endophytic bacterial colonization and plant defense induction are still under investigation. Several management strategies have been developed to prevent and control GTD, including pruning, trunk renewal, and chemical treatments. However, these strategies have limitations, and a sustainable and eco-friendly alternative is required [6].

*Calonectria pseudonaviculata* (Cps) is a fungal pathogen that infects several plant species, including *Buxus* (boxwood), *Pachysandra* (pachysandra), and *Sarcococca* spp. (sweet box). However, the mechanism of this pathogen’s adaptation to different host plants has been undefined until recently. Kong et al. [9] conducted a series of experiments to understand the adaptation process of Cps to its hosts, by measuring three components of aggressiveness: infectibility, lesion size, and conidial production.

In this study, the authors performed serial passage experiments using the three host plants, and measured changes in Cps aggressiveness components. Detached leaves of individual hosts were inoculated with isolates from the originating host (P0), followed by nine serial inoculations of new leaves of the same host with conidia from the infected leaves of the previous inoculation. The results indicated that all boxwood isolates maintained their capability of infection and lesion expansion through the ten passages, whereas most non-boxwood isolates lost these abilities during the passages. Isolates from plants of origin (-P0), and their descendants isolated from passages 5 (-P5) and 10 (*-P10), were used to evaluate changes in aggressiveness toward all three hosts with cross-inoculation [9].

Interestingly, post-passage boxwood isolates presented enlarged lesions on the pachysandra, while sweet box P5 and pachysandra P10 isolates showed reduced aggressiveness toward all hosts. These results suggest that Cps is most adapted to boxwood and less adapted to both sweet box and pachysandra. Therefore, Cps appears to be undergoing speciation, with the fastest coevolutionary pace with the hosts with boxwood, intermediate with sweet box, and the slowest with pachysandra. These findings shed light on the adaptation process of fungal pathogens and highlight the importance of understanding host–pathogen coevolutionary dynamics. The results also have implications for the management of plant diseases caused by Cps. Boxwood is a valuable landscape shrub, and the discovery of Cps as most adapted to boxwood underscores the importance of effective disease management practices for this plant species. Contrastingly, pachysandra and sweet box may be less susceptible to the disease and may require fewer management interventions [9].

Passion fruit *(Passiflora edulis* Sims) is a highly valued fruit crop that is widely grown in tropical and sub-tropical regions for its economic, nutritional, and medicinal properties [10]. However, leaf blight caused by *Nigrospora sphaerica* is an emerging disease that affects the production and quality of passion fruit. *Bacillus* species are commonly used as biocontrol agents and plant-growth-promoting bacteria (PGPB) in agricultural systems. However, little is known about the endophytic existence of *Bacillus* spp. in the passion fruit phyllosphere, and their potential as biocontrol agents and PGPB [11,12].

In a recent study conducted by Wang et al. [13], the authors isolated 44 endophytic strains from 15 healthy passion fruit leaves obtained from the Guangxi province, China. Of the 44 strains, 42 were identified as *Bacillus* species. The inhibitory activity of the endophytic *Bacillus* spp. strains against *N. sphaerica* was examined in vitro, and 11 strains were found to inhibit the pathogen by more than 65%. All 11 strains produced biocontrol- and plant-growth-promoting metabolites, including indole-3-acetic acid (IAA), protease, cellulase, phosphatase, and solubilized phosphate. Furthermore, the researchers tested the plant growth promotion traits of the 11 endophytic *Bacillus* strains on passion fruit seedlings. One strain, coded *B. subtilis* GUCC4, significantly increased passion fruit stem diameter, plant height, leaf length, leaf surface, fresh weight, and dry weight. In addition, *B. subtilis* GUCC4 reduced the proline content, which indicated its potential to positively regulate passion fruit biochemical properties, and resulted in plant-growth-promotion effects. Finally, the biocontrol efficiencies of *B. subtilis* GUCC4 against *N. sphaerica* were determined in vivo, under greenhouse conditions. *B. subtilis* GUCC4 was found to significantly reduce disease severity, similar to the fungicide, mancozeb, and a commercial *B. subtilis*-based biofungicide.

The fruit rot disease (FRD) affecting arecanut crops in India has been thoroughly addressed by Patil et al. [14]. The authors aimed to evaluate the efficiency of different treatments to control the disease, and to estimate the yield response and returns on investment. They analyzed data collected from 21 field trials, conducted in five arecanut-growing regions in India between 2012 and 2019. The results showed that the spraying of fungicides containing different active ingredients or formulations proved to be the most effective preventative measure to control FRD, followed by treating palms with soil microbial consortia or commercial formulations of organic fungicides. These treatments resulted in significant yield increases in arecanut production. The authors suggested that the implementation of such treatments by arecanut growers could help sustain a maximum yield of their crops.

To better understand the spatiotemporal dynamics of fruit rot and its relationship with climate in arecanut-growing areas, Patil et al. [15] conducted a study in three major growing regions of Karnataka, India. The study monitored 27 sampling sites for two consecutive years and assessed the percentage disease intensity (PDI) on 50 randomly selected palms. The researchers employed the ordinary kriging (OK) technique to predict disease occurrence at unsampled locations. Results indicated that the disease intensity was substantial in the Malnad and coastal regions, with significant spatial clusters across the studied regions. The temporal analysis indicated significant variation in PDI, with disease initiation occurring early in the season in the Malnad and coastal regions compared to the Maidan region. Additionally, the study established that temperature, relative humidity, and total rainfall were positively associated with disease occurrence. Regression model analysis revealed that the association between maximum temperature, relative humidity, and total rainfall with PDI was statistically significant.

The sudden appearance of the Mediterranean pine engraver (*Orthotomicus erosus*) poses a significant pest threat in Croatia. This beetle was detected in 2017 in Marjan Forest Park, and quickly spread throughout the Dalmatian coast. Kovač et al. [16] aimed to investigate the relationship between the pest and fungal infection that caused severe blue staining in the sapwood of attacked pine trees. Isolates were obtained from the beetles, their galleries, and blue-stained sapwood, and identified according to their morphological characteristics and DNA sequencing. The results determined the identification of six different Ophiostomatales fungi, confirming the first record of Ophiostomatales as organisms associated with *O. erosus* and pine species in Croatia.

The post-harvest brown rot disease caused by *Monilinia fructigena* is a significant issue in nectarine production. Lyousfi et al. [17] evaluated the simultaneous treatment of antagonistic bacteria *Bacillus amylolquefaciens* (SF14) and *Alcaligenes faecalis* (ACBC1) with the food additive sodium bicarbonate (SBC), to control the disease and its effect on the post-harvest quality of nectarines. Four concentrations of SBC were tested, and the results showed that the bacterial antagonists were compatible with different SBC concentrations, and their viability was not affected. In vitro and in vivo bioassays showed that the treatments had a strong inhibitory effect on the disease, with a significant improvement in their biocontrol efficacies when combined. The combination of bacterial antagonists with SBC revealed a significant reduction in disease severity, ranging from 9.27% to 64.83%, compared to the untreated control. The disease incidence and lesion diameter in fruits treated with bacterial antagonists alone, or in combination with SBC, were significantly lower than those in the untreated fruits. In addition, the treatment did not affect the appearance, firmness, total soluble solids, and titratable acidity of the fruits, indicating that the treatment did not compromise the post-harvest quality of the nectarines. The results suggested that the improved disease control by the two antagonistic bacteria was likely due to the additional inhibitory effects of SBC on the mycelial growth and spore germination of the pathogenic fungus [17].

To investigate the temporal dynamics of shot hole disease (SHD) and its management in an integrated plum orchard, Molnár et al. [18] monitored the progress of SHD over three years under four different training systems and on four different plum cultivars. The study aimed to identify the periods when training systems and cultivar combinations had the ability to reduce SHD development. The results showed that both SHD incidences and the area under the disease progress curves (AUDPC) were significantly affected by the training system, cultivar, and year. Cultivars with high or mid-high susceptibility to SHD showed continuous SHD development from May to November, while cultivars with low susceptibility showed no symptoms until mid-summer, and then progressed slowly until November. The high-density (4 × 1.5 m) training system consistently reduced SHD incidence and AUDPC for three cultivars (‘Čačanska lepotica’, ‘Stanley’ and ‘President’) in September, October, and November, compared to the low-density (6 × 3 m) training system. However, cv. ‘Bluefre’ showed no effect on disease incidence or AUDPC, due to very high disease incidences in all training systems from September to November [18].

The study established by Gulati et al. [19] investigated the potential use of *Leucaena leucocephala* leaves as green manure, and identified the fungal species responsible for their decomposition. The study was motivated by the necessity to increase global agricultural output by 70% by the year 2050, to address international food security concerns, while also addressing the negative impacts of current agricultural techniques on the environment and human health. The maintenance of good-quality soil organic matter, particularly in tropical countries such as India, requires a steady input of organic residues to maintain soil fertility. This study involved the isolation and identification of 52 different species of fungi from *L. leucocephala* leaves at various stages of decomposition. The percentage of fungal occurrences increased as the leaves senesced and finally decomposed, with a higher occurrence of Deuteromycetes (75.47%) and a lower rate of Ascomycetes (9.43%) during almost all decomposition stages. Basidiomycetes such as unidentified sclerotia and *Rhizoctonia solani* showed a gradual increase as the leaves senesced and finally decomposed. *Didymium nigripes* was the only Myxomycete isolated from completely decomposed leaves in the moist chamber.

Interestingly, it has been found that *L. leucocephala* leaves have potential use as green manure due to their high fungal diversity and their ability to provide organic residues which maintain soil fertility. The study also highlighted the importance of monitoring the fungal colonization of *L. leucocephala* leaves during the decomposition process, to identify the fungal species responsible for decomposition and understand the dynamics of nutrient cycling in the soil. Moreover, the study found that wet seasons had more fungi on average than dry seasons [19].

Kazakhstan, the fourteenth largest wheat producer globally, lacks a comprehensive survey of wheat root and crown rot, despite its importance as a crop disease. A quantitative survey was conducted to determine the distribution of fungi associated with root and crown rot of wheat (*Triticum* spp.) [20]. Samples were collected from the roots and stem bases of affected plants during the 2019 growing season. A total of 1221 fungal isolates were obtained from 65 sites across the central, eastern, and southeastern parts of the country. Morphological and molecular tools were used to identify the species of fungal isolates, and internal-transcribed spacer (ITS), translation elongation factor 1-alpha (EF1-α), and glyceraldehyde-3-phosphate dehydrogenase (GAPDH) sequences were successful in identifying the fungal species. The results showed that *Bipolaris sorokiniana* and *Fusarium acuminatum* were the most prevalent fungal species isolated, present in 86.15% and 66.15% of the surveyed fields, respectively. *F. pseudograminearum*, *Fusarium* sp., *C. spicifera*, and *C. inaequalis* were identified as pathogens of wheat for the first time in Kazakhstan.

Seventy-four isolates, representing 16 species, were tested via inoculation on the susceptible *T. aestivum* cv. Seri 82, and the results showed that *F. culmorum*, *F. pseudograminearum*, *B. sorokiniana*, *Fusarium* sp., *R. solani*, *F. redolens*, *C. spicifera*, *C. inaequalis*, and *N. oryzae* were virulent. The study demonstrates the presence of a diverse spectrum of pathogenic fungal species relevant to the wheat crown and root rot in Kazakhstan. The results will aid in developing effective strategies for controlling the spread of wheat root and crown rot, which will be beneficial to the wheat industry in the country. Furthermore, the identification of *F. pseudograminearum*, *Fusarium* sp., *C. spicifera*, and *C. inaequalis*, as pathogens of wheat in Kazakhstan, may lead to further studies on the distribution and prevalence of these species within wheat crops globally [20].

To conclude, the study of plant and tree pathogens is an important area of research, with significant implications for agriculture, forestry, and the environment. Isolating and characterizing pathogens is crucial for understanding their biology and developing effective control strategies. These strategies depend on various factors, such as the type of pathogen, the host plant, and the environment. Chemical control methods, such as fungicides and bactericides, can be effective; however, they have drawbacks, such as toxicity to humans and the environment, and the development of resistance. Biological control methods using beneficial microorganisms or natural enemies of the pathogen are gaining more attention as a safer and sustainable alternative. Nevertheless, it is important to note that prevention is often the most effective strategy for managing plant and tree pathogens. This includes measures such as selecting disease-resistant plants, crop rotation, and sanitation practices. In addition, early detection and rapid response to new pathogen outbreaks are crucial in preventing their spread and minimizing their impact.

As the world population continues to grow and the demand for food and wood products increases, further exacerbated by challenges arising from climate change, the study of plant and tree pathogens will become even more crucial. Continued research and development of new control strategies will be necessary to ensure sustainable and healthy agriculture and forestry systems.

The papers presented in this Special Issue concern different aspects of the interrelations between different crops and pathogens, and the authors hope this will prompt budding researchers to work in this field of research.
